# Prediction of Medical Concepts in Electronic Health Records: Similar Patient Analysis

**DOI:** 10.2196/16008

**Published:** 2020-07-17

**Authors:** Nhat Le, Matthew Wiley, Antonio Loza, Vagelis Hristidis, Robert El-Kareh

**Affiliations:** 1 Department of Computer Science & Engineering University of California, Riverside Riverside, CA United States; 2 School of Medicine University of California, Riverside Riverside, CA United States; 3 Department of Medicine University of California, San Diego San Diego, CA United States

**Keywords:** consumer health information, decision support techniques, electronic health record

## Abstract

**Background:**

Medicine 2.0—the adoption of Web 2.0 technologies such as social networks in health care—creates the need for apps that can find other patients with similar experiences and health conditions based on a patient’s electronic health record (EHR). Concurrently, there is an increasing number of longitudinal EHR data sets with rich information, which are essential to fulfill this need.

**Objective:**

This study aimed to evaluate the hypothesis that we can leverage similar EHRs to predict possible future medical concepts (eg, disorders) from a patient’s EHR.

**Methods:**

We represented patients’ EHRs using time-based prefixes and suffixes, where each prefix or suffix is a set of medical concepts from a medical ontology. We compared the prefixes of other patients in the collection with the state of the current patient using various interpatient distance measures. The set of similar prefixes yields a set of suffixes, which we used to determine probable future concepts for the current patient’s EHR.

**Results:**

We evaluated our methods on the Multiparameter Intelligent Monitoring in Intensive Care II data set of patients, where we achieved precision up to 56.1% and recall up to 69.5%. For a limited set of clinically interesting concepts, specifically a set of procedures, we found that 86.9% (353/406) of the true-positives are clinically useful, that is, these procedures were actually performed later on the patient, and only 4.7% (19/406) of true-positives were completely irrelevant.

**Conclusions:**

These initial results indicate that predicting patients’ future medical concepts is feasible. Effectively predicting medical concepts can have several applications, such as managing resources in a hospital.

## Introduction

### Background

Medicine 2.0—the intersection of Web 2.0 and health care services, apps, and tools—brings new opportunities for patients to actively contribute to their own care [1]. With the rapid adoption of patients’ electronic health records (EHRs) [2], allowing users to find patients with similar experiences and health conditions based on their EHR has the potential to improve the quality of care and expand options for health care solutions [3]. This approach may lead to novel apps for patients, such as self-management recommendations based on big data aggregation across cohorts [4]. Apps that allow patients to find, discuss, and share health data and information can improve patient outcomes while raising meaningful discussions in disease management [5]. Therefore, finding patients with similar experiences and health conditions is a critical step for patients to contribute to their own care. This capability is becoming more important as more patient records become available (with user consent and commonly anonymized), for instance, through health social networks that aim to connect patients, which drive the need for patient-centered health informatics [6,7].

We evaluated the *hypothesis* that we can predict possible future medical concepts in a patient’s EHR by leveraging the EHRs of other patients in the collection. Medical concepts are entities of a medical ontology, which is a knowledge network of medical concepts, where concepts and their definitions are categorized and interconnected (normally via a hierarchy) to present their semantic meanings. Given a point of time, a patient’s current medical history is stored in form of EHRs. Future medical concepts are defined as the ones appearing in the patient’s EHRs after that point, which is also the patient’s future medical record. To evaluate our hypothesis, we first organized each patient’s EHR in the database as a list of chronological medical events, which can be divided into a prefix (a sequence of events up to a time moment) and a suffix (a sequence of events that happened after this time moment). Then, we used various interpatient similarity measures to locate other patients’ EHRs that have prefixes similar to the current patient’s EHR. Finally, we processed the time-based suffixes of the matched EHRs to determine which medical concepts are probable for the future of the current patient’s EHR. In short, our method uses EHRs of patients with similar past medical developments to predict a patient’s upcoming developments.

Furthermore, our method offers the prediction’s explanation by providing similar patients and medical concepts influencing the prediction; thus, it does not suffer the interpretability limitation of common deep learning techniques [8]. Although we used the Multiparameter Intelligent Monitoring in Intensive Care (MIMIC) II database to evaluate our methods, our methods are applicable to any database of EHRs, where a set of medical concepts can be extracted for various time instances (eg, hospital visits) during a patient’s care.

Patients are not the only stakeholders who stand to benefit from the prediction of future medical concepts in an EHR; clinicians and clinical researchers can also benefit from a what-if analysis based on similar patients. For example, when a physician is answering questions for a patient or the patient's family, such an analysis may be helpful as supporting evidence, especially to provide data-driven guidance in the absence of specific gold standard [7]. Moreover, the clinician may view the changes in the probable future EHR of a patient if a specific therapy is undertaken. From a research standpoint, clinical researchers may be interested in finding patients with similar predicted concepts when performing nonrandomized studies, for example, for matching cases and controls.

### Related Work

Research related to our study is divided into 2 groups: those that consider (1) interpatient similarity measures and (2) analysis and prediction via aggregated patient data. The former is related to patients with similar experiences, and health conditions were used for predicting future medical concepts. The latter group is related in that an aggregate of patient data across a database of EHRs was used for predicting future medical concepts. However, none of the related studies have defined the notion of EHR prefixes and EHR suffixes when aggregating patient data or finding patients with similar experiences and health conditions.

### Interpatient Similarity Measures

When measuring patients with similar experiences and health conditions, we leveraged previous papers, which have studied several interpatient distance functions. Methods include case-based reasoning, vector space models, bag-of-concepts (BoCs), information content, path length between concepts, common ancestors of concepts, and combinations of these. None of these methods have been applied to EHR prefixes and EHR suffixes for predicting future medical concepts. Thus, the intuitive question is, “Are these interpatient similarity measures powerful enough to identify patients with similar histories and futures?”

Cao et al [9] used case-based reasoning to find patients with similar experiences and health conditions based on clinical text. They found that medical concepts are superior features compared with a bag-of-words approach. Similar to this study, the authors restricted medical concepts to a specific subset of semantic types, but the authors did not consider semantic similarity between concepts—for example, 2 concepts may be neighbors in the Systemized Nomenclature of MEDical Clinical Terms (SNOMED-CT) ontology—when comparing patients. Mabotuwana et al [10] studied an ontology-based similarity measure for radiology reports where the authors extended cosine similarity to include the semantic similarity of medical concepts mentioned in radiology reports. The authors found that the addition of semantic similarity allows a vector space model to differentiate between radiology reports of different anatomical and image procedure–based classes. Plaza and Diaz [11] studied concept graphs for measuring interpatient similarity. Given a set of concepts for a patient, all ancestors of each concept are retrieved and assigned a weight based on their depth, where deeper concepts have higher weights. This method is studied in this study and explained in greater detail in the Methods section. Melton et al [12] studied a variety of interpatient distance measures, including BoCs and average path length (APL). Both the BoCs and unweighted APLs are investigated and described in greater detail in the Methods section.

### Analysis and Prediction of Aggregated Patient Data

Related work on aggregating patient data for analytics employs a patient database to provide recommendations, analysis, and/or predictions. Gotz et al at IBM Corporation [13-15] developed an interactive system to aid domain experts in retrospective patient cohort analysis. Similar to our study, their system finds a cohort of patients with similar health conditions based on the EHR of the physician’s current patient via symptoms. Statistics for the cohort are aggregated and visualized using a variety of techniques, including an outflow graph that models the evolution of symptoms over time and the respective outcomes. Unlike this study, their system does not predict future medical concepts, nor do they use ontologies when measuring patients with similar health conditions. However, their study complements our study in that the user can use predicted symptoms to explore possible outcomes in the outflow graph.

PatientsLikeMe has also examined the effects of aggregating patient data [4,16]. A web-based survey found that users reported several benefits from having access to aggregated patient statistics. Furthermore, they found a correlation between perceived benefit and the number of website features used by a user, along with demographic similarities between the users of the web-based platform and actual patient populations. This study aimed to complement the data created by PatientsLikeMe by employing aggregated data to predict future medical concepts.

Recent advancements in deep learning offer a new, powerful predictive tool for patients’ EHRs [17]. Miotto et al [18] proposed a 3-layered stack of denoising autoencoders to learn a vector representation of each patient from an EHR database of approximately 700,000 patients and then used this *deep patient* embedding to predict the probability of patients developing 78 diseases. Studies by Razavian et al, Lipton et al, Choi et al, and Nguyen et al [19-22] explored the temporal order of medical events and different neural network architectures, such as recurrent convolutional networks. Rajkomar et al [23] represented a patient’s entire EHR as a temporal sequence of medical events in the fast health care interoperability resources format and applied various deep learning models to learn the patient’s representation for further predictions: inpatient mortality, 30-day unplanned readmission, long length of stay, and 14,025 International Classification of Diseases-9th revision, diagnosis codes. In general, these methods learn the patient’s vector representation, which is used to model downstream prediction tasks such as classification or regression problems. Although these studies restrict their predictions to a predefined medical concept set, our study makes predictions of any medical concepts appearing in patients with similar health conditions. Moreover, whereas deep learning approaches offer limited interpretability [8], our method explains how a prediction is made.

## Methods

We represented each patient as a set of medical concepts from SNOMED-CT [24]. We extracted medical concepts using the MetaMap library [25]. Then, to identify patients with similar health conditions, we adopted various distance functions studied in the literature [11,12]. We showed how to extend these distance functions to predict future medical concepts, given a query patient. We demonstrated and evaluated these methods on the MIMIC II clinical database, which contains patient data from visits to an intensive care unit (ICU) [26].

### Framework and Method for Predicting Future Concepts Using Similar Patients

**Figure 1:**
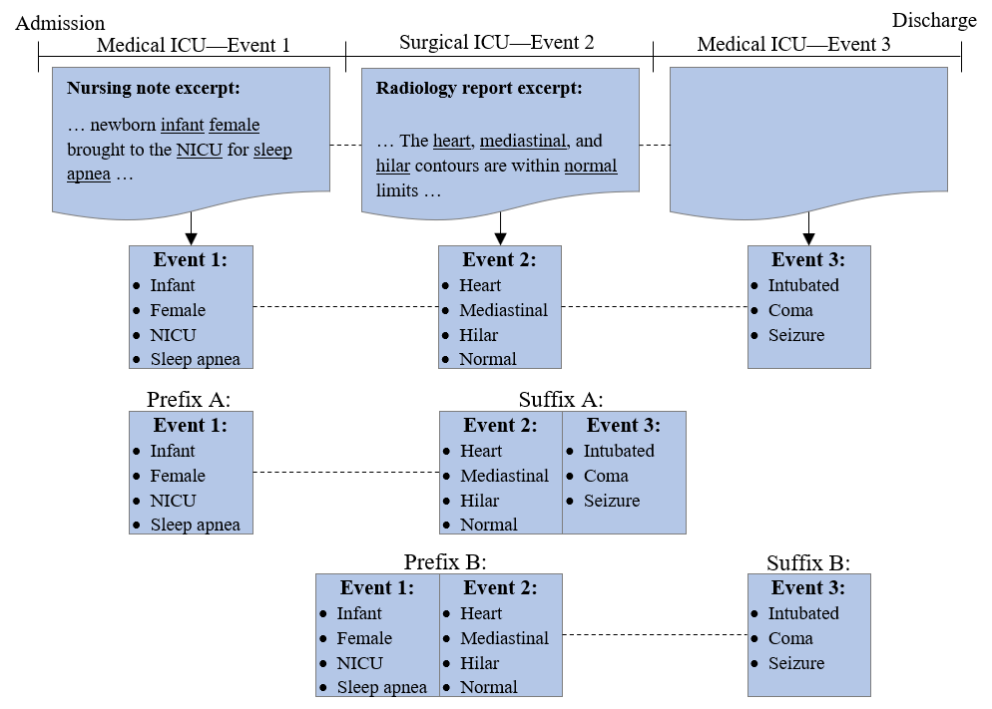
An example of a patient visiting the intensive care unit, discretized by ward transfers. In this example, the patient was admitted to the medical intensive care unit, transported to radiology, and transferred to the surgical intensive care unit. As this example contains 3 events, there are 2 possible electronic health record prefixes and 2 possible electronic health record suffixes. ICU: intensive care unit; NICU: neonatal intensive care unit.

First, we proposed our framework for discretizing EHRs into events, yielding the notion of *EHR prefixes* and *EHR suffixes*. Consider a database of patient visits to an ICU. One possible method to discretize these visits is to exploit transfers between wards within the ICU, as illustrated by the example in Figure 1. In this example, the patient is admitted to the medical ICU, transferred to the surgical ICU, and then transferred back to the medical ICU. The patient’s time in each ward represents a distinct *event*, where clinical notes are recorded that report the patient’s status; thus, medical concepts reported in each ward are associated with a specific event. Furthermore, these events have a natural ordering, which produces the notion of EHR prefixes and EHR suffixes. In this example, there are 2 possible EHR prefixes, *[Event1]* and *[Event1, Event2]*, and 2 possible EHR suffixes, *[Event2, Event3]* and *[Event3]*. Hence, each EHR prefix and EHR suffix is associated with a set of medical concepts, as shown at the bottom of Figure 1.

The motivation for discretizing EHRs into events is that health care changes over time with respect to medical conditions, procedures, findings, and drugs observed from the past. Given a new patient, our goal is to find similar EHR prefixes from the EHR database such that the respective EHR suffixes will predict the new patient’s future. Let the new patient’s EHR be denoted by *Q*, where *Q* is represented as a set of medical concepts defined on an ontology. Let *Q*^p^_k_ represent the set of medical concepts obtained from the first *k* events, where the superscript *p* denotes that this set is an EHR prefix. The corresponding EHR suffix is denoted by *Q^S^_k_*_+1_, which represents the set of medical concepts from event *k+1* to the last event in the EHR. Note that in a clinical setting, we would use the whole EHR as *Q*^p^_k_ as the goal is to predict future concepts, given the current state of the patient. Finally, let *D* be the database of records within the EHR. We now define our concept prediction algorithm that consists of 2 steps: (1) finding similar records and (2) returning concepts with high confidence.

#### Concept Prediction Algorithm

##### Step 1: Compute Similar Electronic Health Record Prefixes

In particular, find the set *S* of EHR suffixes that correspond to the EHR prefixes *P_i_* in *D* whose dissimilarity with respect to *Q^p^_k_* is less than some *dissimilarity threshold τ*: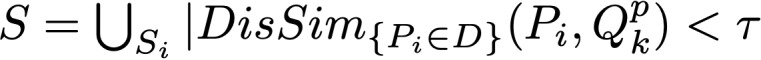 where *P_i_* is an EHR prefix of events from a single visit, *S_i_* is the corresponding EHR suffix, and *DisSim* is an interpatient dissimilarity function. Note that we only considered the most similar EHR prefix for each visit.

##### Step 2: Return Concepts With High Confidence

Let 
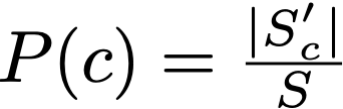
 be the confidence of concept c, where *S'_c_* is the EHR suffixes from *S* that contain *c*. We return 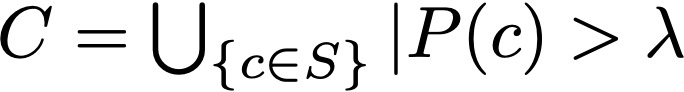, which is the set of concepts in *S* with confidence greater than the *confidence threshold 𝜆*.

**Figure 2:**
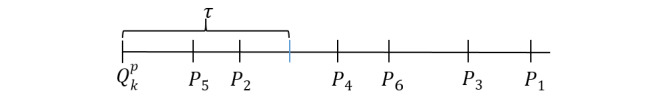
Dissimilarities of electronic health record prefixes with respect to the k-events prefix of a patient Q denoted by Q_k^p^.

Figure 2 illustrates step 1 of the concept prediction algorithm, where only prefixes *P_2_* and *P_5_* have dissimilarities from the query prefix *p* (or with respect to *Q*^p^_k_) smaller than the threshold τ; thus, their corresponding suffixes *S_2_* and *S*_5_ are included in *S*. Define 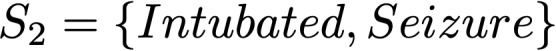. Furthermore, let *P*_5_ and *S*_5_ be EHR prefix B and EHR suffix B from Figure 1. Thus, 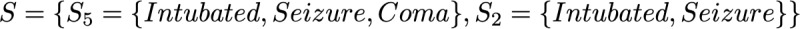. Let λ=0.7, then step 2 of the algorithm returns *C*={*Intubated, Seizure*}.

Hence, we can evaluate both parameters and *DisSim* using traditional measures of specificity, sensitivity, and precision. Let, *U* be the universe of all medical concepts. True-positives (TPs), true-negatives (TNs), false-positives (FPs), and false-negatives (FNs) are defined by:


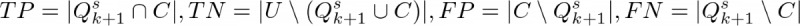


We have also extended our definitions of TP, TN, FP, and FN to consider *fresh concepts* only. Fresh concepts are concepts that appear in the query EHR suffix, 
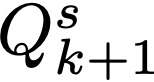
, which do not appear in the query EHR-prefix, 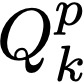. We argue that fresh concepts are more challenging and have a higher potential to be clinically useful for prediction. We analyzed fresh concepts separately from all concepts as concepts that appear in the query EHR prefix are likely to persist into the suffix and thus would skew our evaluation of fresh concepts. Therefore, we ignore concepts that appear in 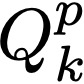 when evaluating any measures concerning TP, TN, FP, or FN.

**Figure 3:**
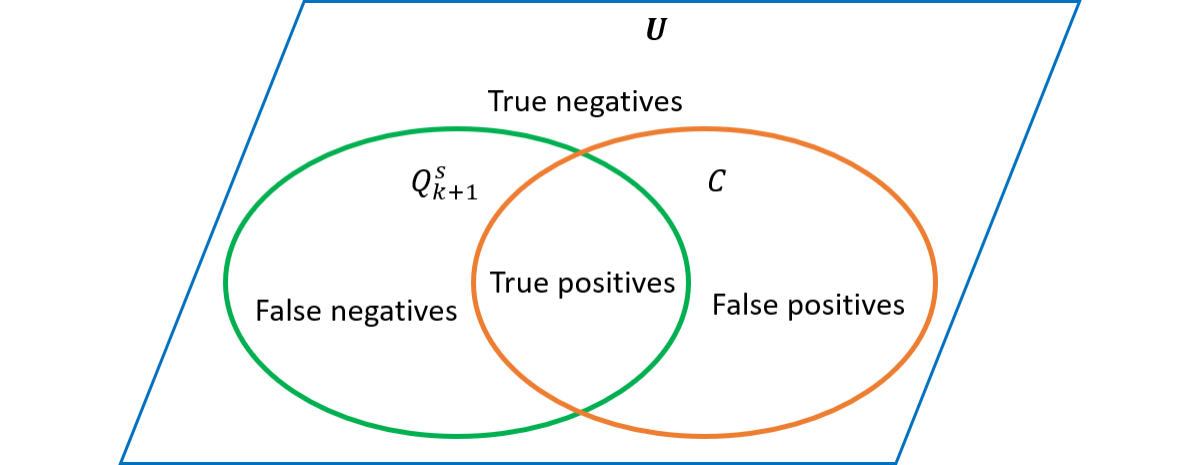
The connection between the ground truth concepts and the predicted concept space.

Figure 3 illustrates the connection between the entire set of concepts *U*, the predicted set of concepts *C*, and the ground truth 
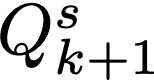
. In our experiments, the size of 
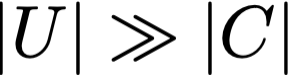
, and thus, the number of TNs skews the value of specificity. Therefore, we assessed the parameters and interpatient distance measures using the harmonic mean of sensitivity and precision, commonly known as the F-measure in information retrieval.

### Interpatient Distance Measures

We evaluated 4 interpatient dissimilarity measures proposed in the literature [4,5]: (1) *BoC*, (2) *CAs*, (3) *APL*, and (4) symmetric APL (*APL_SYM*).

Let *A* and *B* be the sets of medical concepts.

For BoC, the dissimilarity between *A* and *B* is defined as the sum of the number of concepts that appear in A but not in B and in B but not in A, divided by the size of their union [5]. union of *A* and *B* is also a set, and therefore, the size of the union only considers each concept once:


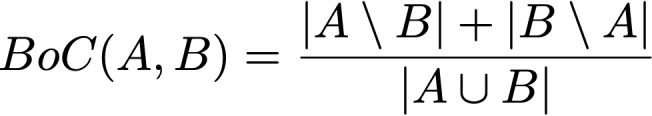


BoC produces values between 0 and 1, where 0 represents maximum similarity, and 1 represents minimum similarity. Note that BoC is symmetric; hence, *BoC*(*A, B*)=*BoC*(*B, A*).

In CA, for each concept, for each concept c_a_ in A, we retrieved all ancestor concepts in the concept hierarchy and assigned to each concept and its ancestors a weight, where each c_a_ is assigned a weight of 1, and ancestors of each c_a_ are assigned a weight relative to their distance from c_a_. An analogous weighting procedure is applied to all concepts and their ancestors in *B*. Weights are averaged if a node is assigned more than one weight.

Let *A'* and *B'* be the set of concepts and their ancestors for *A* and *B*, respectively. When computing the dissimilarity from *A* to *B*, we examined each concept in *A’* and check if it exists in *B’*. If it exists, the given concept in *A’* is assigned a value equal to its own weight, and zero otherwise [4]:

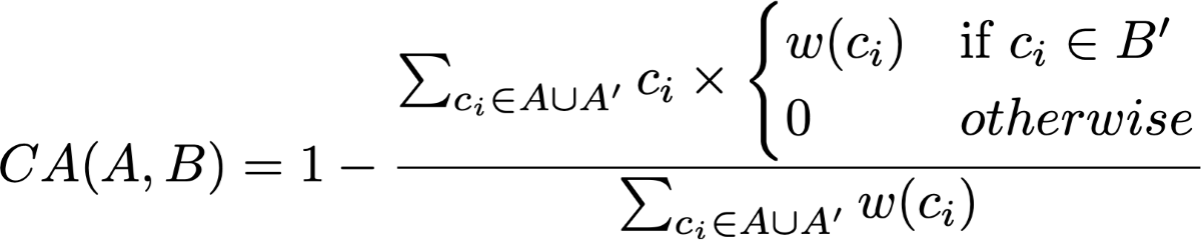

where *w (c_i_)* is the weight assigned to the concept *c_i_*. Hence, the abovementioned sum measures the overlap between the concepts and the ancestors of *A* and *B*. Scores from CA range from 0 to 1, where a score of 0 represents maximum similarity, and 1 represents minimum similarity. By definition, CA is not symmetric.

The APL measure finds the minimum number of edges between each concept in A with every concept in B. APL sums the distances across all concepts in A to obtain the dissimilarity of *A* to *B* [5]:


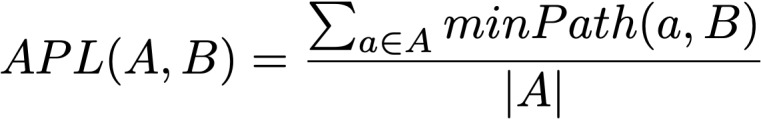


A score of 0 implies a maximum similarity. By definition, APL is not symmetric; APL_SYM is the sum of *A* to *B* and *B* to *A*:


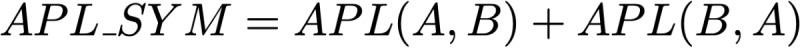


### Preparation of Multiparameter Intelligent Monitoring in Intensive Care II Data Set

We applied our framework and the aforementioned interpatient dissimilarity measures to the MIMIC II clinical database—a database of EHRs collected over a 7-year period from multiple ICUs at a medical center in Boston [26]. Several types of clinical notes are recorded during a visit, including radiology reports, nursing notes, and physician notes. We parsed each note to extract medical concepts from the clinical text. Each note is associated with a timestamp that represents its creation time. We used these timestamps to map notes to events, defined as ward transfers, generating a list of concepts for each event.

First, we parsed medical concepts from each type of note using the MetaMap library [25]. Before parsing each note, abbreviations such as *OMG* were identified and expanded using an abbreviation list similar to the list of Wiley et al [27]. The MetaMap library maps free text to biomedical concepts are defined in the Unified Medical Language System (UMLS) [28]. Each concept in the UMLS corresponds to one or more semantic types [29], which further maps to semantic groups [30]. Previous studies have shown that disorders, physiology, chemicals and drugs, procedures, and anatomy are the most important UMLS semantic groups when measuring interpatient similarity [11]. Negated concepts are identified via MetaMap, and these concepts are ignored, as previous work has shown that absent concepts are not relevant to patient similarity [11]. After obtaining a list of relevant concepts, each concept from the UMLS is converted to a concept from SNOMED-CT using the MRCONSO table [31].

A single patient visit may consist of several transfers between wards. Each of these transfers is considered to be a *census event* in the MIMIC II database. The rationale for this definition of an event is that each time a patient enters a new care unit, there may be a significant change in the patient’s status, for example, the patient’s condition worsened, and he was transferred to the surgical ICU.

If a patient visits a hospital multiple times, each visit is treated independently, that is, multiple visits are viewed as different patients for the purpose of our similarity matching algorithm. This decision is not critical for the MIMIC II data set because a majority of patients only have one visit. Related work has shown that the abovementioned concept of census events provides an effective timeline of a patient’s record, where concepts within an event are semantically associated with each other [32].

### Computation Time Analysis

The computation cost to extract ancestors is linear with respect to the number of ancestors. As the ontology is a wide directed acyclic graph (DAG) instead of a deep one, each concept has up to 61 ancestors, and 29 ancestors on average. We used Dewey encoding to speed up both the retrieval of ancestors and calculation of concept distance. In particular, a concept’s Dewey encoding encapsulates its ancestor information, for example, if concept *C2315591* is encoded as *$.8.96.45*, this implies that the concept’s ancestors are *$.8* and *$.8.96*. Using Dewey [33] encodings, the distance between 2 concepts is reduced to be a string comparison between their encodings; that is, we computed the distance between the concepts and their lowest common ancestor, which again has cost linear on the DAG depth.

## Results

### Anecdotal Example

We started with a real anonymized example from the MIMIC II dataset to demonstrate the potential utility of our approach. Bob was involved in a motor vehicle collision where he struck his head and lost consciousness. He arrived at the medical ICU with a chief complaint of severe shoulder pain and bleeding from his nostrils. After arriving at the medical ICU (event 1), Bob was transferred to the surgical ICU for further care (event 2). During his stay in the surgical ICU, the staff observed symptoms of pneumonia and pulmonary aspiration. Bob was then transported to radiology (event 3), where tests revealed that Bob indeed had both pneumonia and pulmonary aspiration. We executed our prediction method using event 1 as a query. In particular, we used *CA*, with τ=0.5 and λ=0.3. Of the suffixes of patients with similar EHR prefixes, 50% contain the concepts of pneumonia and pulmonary aspiration, whereas 29% and 23% of all patients in the general ICU population contained the concepts of pneumonia and pulmonary aspiration, respectively.

### Event-Based Analysis of the Multiparameter Intelligent Monitoring in Intensive Care II Data Set

We only considered visits with more than one event because visits with 1 event cannot be split into EHR prefixes and EHR suffixes. In total, there are 4083 visits over 3971 unique patients; thus, patients with multiple visits account for less than 3% of the total number of visits. Visits with 2 events dominate the data set, accounting for 80% of the total visits, whereas visits with 3 events accounted for 15% of the total visits. In general, a longer visit produces more medical concepts, implying that new concepts are found as the patient’s visit progresses. Visits of length 2, 3, and 4, respectively, have 291, 434, and 539 unique medical concepts on average. The corresponding number for visits of more than 4 events is 725. On average, each event contains 187 medical concepts, and each visit contains 325 medical concepts. Furthermore, these concepts are dominated by disorders (36%) and procedures (22%). The other concept semantic groups are anatomy (20%), drugs (12%), and physiology (10%).

### Prediction Results

We evaluated the interpatient distance measures BoC, CA, APL, and APL_SYM on the aforementioned admissions of the MIMIC II database using our framework of EHR prefixes and EHR suffixes. Our first objective was to tune the parameters τ and λ using the *F* measure. We split the admissions into training and testing datasets, where 20% of the admissions were used for training, and 80% of the admissions were used for testing. Table 1 reports the combination of τ and λ that produced the highest *F* measure for each interpatient distance measure using the training data set. APL_SYM obtains the highest *F* measure, precision, and sensitivity, whereas APL obtains the highest specificity.

**Table 1:** The best parameters for each distance function based on the training data set.

*DisSim*	τ	λ	*F* measure (%)	Specificity (%)	Sensitivity (%)	Precision (%)
Bag-of-concept	0.7	0.08	51.8	86.9	52.9	50.6
Common ancestor	0.46	0.25	48.9	94.0	55.2	43.9
Average path length	1.5	0.30	48.7	94.9	52.6	45.4
Symmetric average path length	1.86	0.07	52.4	84.4	52.9	52.0

**Figure 4:**
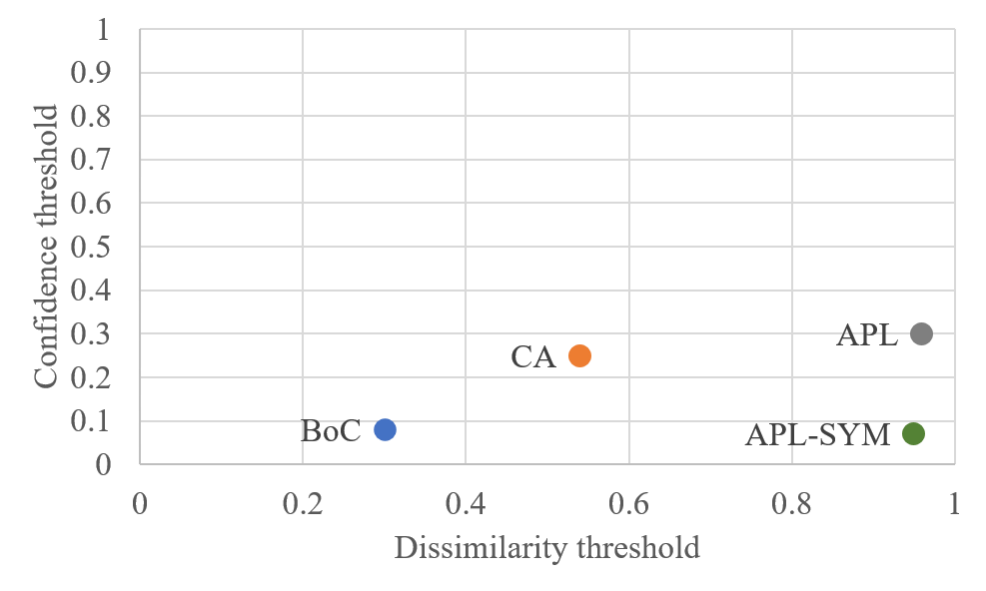
Representation of the optimal choice of the dissimilarity threshold τ and confidence threshold λ for the training data set. APL: average path length; BoC: bag-of-concept; CA: common ancestor; APL-SYM: symmetric average path length.

Figure 4 illustrates a graphical representation of the optimal parameters reported in Table 1, plotting λ on the y-axis and 1−τ on the x-axis. Thus, all concepts from the EHR suffixes of similar EHR prefixes are included with a score to the right of the corresponding vertical dashed line, and from these concepts, all concepts with a confidence above the corresponding horizontal dashed line are included in the predicted EHR suffix. Furthermore, APL and APL_SYM have been normalized by the maximum possible similarity score, where the maximum similarity score is defined as the maximum path length in SNOMED-CT. As shown in this figure, CA and BoC have larger values of dissimilarity compared with APL and APL_SYM. The tightest bounds for both thresholds are for APL and APL_SYM, and the loosest bound is for BoC. This is expected, as the average scores for BoC, CA, APL, and APL_SYM are 0.86, 0.31, 0.07, and 0.07, respectively. Moreover, APL and CA have tightest bounds on the confidence threshold; this is an interesting point, as APL and CA are antisymmetric, implying that *symmetric interpatient distance measures require less confidence when predicting future medical concepts*.

Table 2 reports the results on the testing dataset using the optimal set of parameters reported in Table 1 for fresh and not fresh concepts.

**Table 2:** The results for the testing data set separated by semantic group, using the parameters tuned on the training data set for fresh and not fresh concepts.

Semantic group and *DisSim*		*F* measure (%)		Specificity (%)		Sensitivity (%)		Precision (%)
**All concepts**								
	BoC^a^		51.7		87.1		52.6	50.8
	CA^b^		48.9		94.1		55.7	43.7
	APL^c^		48.7		94.8		52.8	45.2
	APL_SYM^d^		*52.3* ^e^		84.2		53.7	50.9
**Disorders**								
	BoC		49.4		87.8		49.8	49.0
	CA		44.7		95.0		48.4	41.4
	APL		44.4		95.7		46.1	42.8
	APL_SYM		*50.7*		85.0		51.8	49.6
**Procedures**								
	BoC		52.2		85.6		53.3	51.3
	CA		48.9		92.6		57.9	42.3
	APL		48.0		93.6		54.0	43.2
	APL_SYM		*52.6*		82.0		54.4	51.0
**Chemicals and drugs**								
	BoC		49.7		89.8		49.1	50.4
	CA		48.5		96.4		49.9	47.1
	APL		48.1		96.9		47.3	48.9
	APL_SYM		*50.2*		87.7		49.8	50.7
**Physiology**								
	BoC		56.6		82.3		57.1	56.1
	CA		57.8		89.5		69.5	49.5
	APL		*58.4*		90.6		67.6	51.4
	APL_SYM		56.9		80.1		57.7	56.1

^a^BOC: bag-of-concept.

^b^CA: common ancestor.

^c^APL: average path length.

^d^APL_SYM: symmetric average path length.

^e^Italicized numbers indicate the best result of the semantic group.

Similarly, Table 3 reports the same results for fresh concepts only; *fresh concepts are concepts that do not appear in the query EHR prefix and, therefore, are fresh to the query EHR suffix*. We categorized each concept into its semantic group and analyzed each interpatient distance measure with all concepts and concepts restricted to a semantic group; anatomical concepts are omitted in this analysis, as predicting an anatomical site, such as lower back, is not useful in a clinical setting.

**Table 3:** The results for the testing data set separated by semantic group, using the parameters tuned on the training data set for fresh concepts only.

Semantic group and *DisSim*		*F* measure (%)	Specificity (%)	Sensitivity (%)	Precision (%)
**All concepts**					
	BoC^a^	43.7	89.6	43.3	44.1
	CA^b^	34.8	95.7	37.7	32.4
	APL^c^	34.5	96.5	35.0	34.0
	APL_SYM^d^	*44.9* ^e^	86.8	45.3	44.6
**Disorders**					
	BoC	42.1	90.0	40.8	43.5
	CA	32.1	96.3	32.9	31.3
	APL	31.8	97.0	30.6	33.1
	APL_SYM	*44.1*	87.3	43.7	44.5
**Procedures**					
	BoC	43.6	88.5	42.7	44.5
	CA	35.6	94.8	39.3	32.5
	APL	34.7	95.6	36.4	33.2
	APL_SYM	*44.7*	85.0	44.6	44.7
**Chemicals and drugs**					
	BoC	38.6	91.8	34.9	43.1
	CA	30.4	97.5	26.6	35.6
	APL	28.7	97.9	23.7	36.3
	APL_SYM	*39.9*	89.7	36.5	44.1
**Physiology**					
	BoC	46.1	86.1	45.2	47.0
	CA	40.9	92.8	45.3	37.3
	APL	41.2	93.8	43.4	39.2
	APL_SYM	*47.2*	83.9	46.8	47.5

^a^BOC: bag-of-concept.

^b^CA: common ancestor.

^c^APL: average path length.

^d^APL_SYM: symmetric average path length.

^e^Italicized numbers indicate the best result of the semantic group.

As shown in Table 2, the symmetric interpatient distance measures outperform the antisymmetric distance measures across all semantic groups, where APL_SYM performs the best; the only exception is physiology. Comparing these results with Table 3 shows that the gap between symmetric and antisymmetric distance measures widens to a 10% difference in terms of *F* measure. That is, *symmetric interpatient distance measures are more predictive of future medical concepts, especially for fresh concepts*. When considering the symmetric measures APL_SYM and BoC, *APL_SYM consistently performs better*, achieving higher rates of sensitivity and precision in every case.

Furthermore, the *antisymmetric interpatient distance measures performed better with respect to specificity* but achieved a lower precision. That is, antisymmetric distance measures predicted fewer concepts overall to achieve higher rates of specificity with lower rates of sensitivity and precision, which is explained by the conservative choice made during the tuning phase. Another interesting point is that all interpatient distance measures observed an increase in specificity for fresh concepts; however, this increase was greatest for symmetric interpatient distance measures. The reason is that the number of FP decreases for fresh concepts, whereas the nonfresh concepts are more frequently predicted to be in the suffix and, therefore, have a higher frequency of FPs.

#### Clinical Significance of the Subset of Predicted Concepts

We further examined 16 individual concepts identified as important by our physician author (RE) in the ICU setting. We focused on the TP cases (correctly predicted mention in the suffix) to validate the prediction’s importance and FN cases (incorrectly predicted no mention in the suffix) to detect possible significant misses. We presented our predictions in a web interface (Table 4), which is basically a table of predicted concepts, the patient’s EHR prefix/suffix and concepts influencing the prediction in highlight.

**Table 4:** Predictions and explanations provided to our medical student and physician authors to label the clinical significance of a prediction.

Patient ID	Predicted concept and time	Prefix at time of prediction	Suffix from time of prediction
22,487	*Bronchoscopy* (3 hours:23 min:0 seconds)	...Resp: RR 16-20 has periods of apnea when asleep... …There is increased density in the right upper lung field with elevation of the minor fissure consistent with developing atelectasis in the right upper lobe…	…*Bronchoscopy* done secondary to low PaO2...

In Table 4, our domain expert is given a prediction, the patient history, and asked to evaluate if the prediction is helpful. Particularly, in the third column (*Prefix at time of prediction*), we presented the patient history up to the point that our system predicts that a concept(s) will appear in future (in the second column *Predicted concept and time*). The last column in Table 4 (*Suffix from time of prediction*) shows events occurring after the prediction time so that our domain expect can judge if the system’s prediction is significant in the sense that the predicted concepts actually affect the patient and the prediction is not trivial, that is, obviously happen, thus no need for prediction. As we focused on the positive cases, the predictions actually appear in the patient’s suffix and thus are highlighted for the domain expert to evaluate.

Our medical student and physician authors manually mark each case with 1 of 4 categories: (1) mentioned and performed; (2) concept mentioned but it is obvious (ie, little value to clinicians); (3) mentioned but only considered by physician, not performed (ie, the clinicians mentioned this concept in the suffix but in the end did not perform the procedure); and (4) mentioned, but out of context (eg, mentioned as part of the medical history of a patient or while describing a similar case). We reported additional metrics such as specificity, sensitivity, FP, and TN of 7 important concepts in the Multimedia Appendix 1, ordered by concept name. We do not count the cases in which a predicted concept occurs in both the patient’s prefix and suffix. Moreover, if a patient history can be divided into multiple prefix-suffix pairs and the algorithm is able to make predictions for a long prefix, not for the shorter prefix, we do not count the case of a shorter prefix as a negative prediction.

#### True-Positive Analysis

Table 5 reports the fine-grained evaluation of TP cases. Note that we only presented predictions of 7 concepts because our algorithm did not predict the remaining 9 concepts. The bronchoscopy concept was successfully mentioned and performed in the suffix 63 of 63 times in a TP category. Bronchoscopy was positively identified with the keywords in the prefix, usually mentioning respiratory symptoms. Compared with bronchoscopy, surgery is a much more invasive procedure that requires consent of the patient and for the patient to be medically cleared for surgery. This caused 215 surgical concepts to be accurately mentioned and performed but have a significant portion mentioned out of context (16 times) or mentioned but only considered and not performed (25 times). Patients have a craniotomy performed for a variety of reasons. One craniotomy in the medical records analyzed was accurately mentioned and performed, but it was not needed to be predicted. The patient undergoing a craniotomy came in after a motor vehicle collision with an obvious facial fracture, thus not needing to predict the craniotomy, as it would be the only way to treat the patient. In summary, most TP predictions are useful. Overall, 13.1% of the predictions are unhelpful, and mostly fall into the surgery concept.

**Table 5:** Expert evaluation of true positive predictions using 4 fine-grained categories.

Concept	Mentioned and performed	Concept mentioned, but is obvious	Mentioned but only considered by physician, not performed	Mentioned, but out of context
Bronchoscopy	63	0	0	0
Cardiac surgery	5	0	0	0
Colonoscopy	1	0	0	0
Craniotomy	9	1	1	1
Dialysis procedure	47	0	6	1
Refractive surgery enhancement	13	0	0	1
Surgery	215	1	25	16

We illustrated how our algorithm offers useful predictions using a TP case example. In patient ID 22,487, a bronchoscopy was successfully predicted in the suffix (Table 4). The patient had a history of coronary artery disease with chest pain and had a triple coronary artery bypass graft performed to alleviate his symptoms before the prefix. In the prefix, our algorithm highlighted (we *highlighted* a concept in the prefix if it is contributing to the prediction of the target concept in the suffix) *effusion* 7 times, *apnea* 6 times, and *increased density* one time, all related to pulmonary pathology. Heparin, a blood thinner, was also highlighted 7 times by our algorithm. The patient’ s respiratory state began to diminish and was eventually placed on a ventilator, as his course in the hospital progressed. Bronchoscopy was accurately predicted and performed on day 3 and hour 23 in the suffix *secondary to low PaO_2_* with small amounts of suctioned thin secretions, and no plugs were found. The accurately predicted concept is interesting, as the patient was initially presented with chest pain–related symptoms treated by intervention through the cardiovascular organ system but was found to have concurrent complications in the pulmonary organ system.

To obtain the full picture, we presented a TP example that is clinically incorrect. In patient 9122, a surgery was predicted in the suffix, but no performance of a surgery in the suffix was found. This patient was a 25-week premature twin baby born by cesarean section. The only mention of surgery in the suffix is an update by a neonatal intensive care unit nurse stating they were *awaiting surgical time for twin*. No surgery was considered or performed for this patient during the suffix and was only being medically managed for being born prematurely. One of the most highlighted words in the prefix used by the algorithm to predict surgery was *bili* with 35 mentions, *bilirubin* had 3 mentions, and *phototherapy* with 20 mentions—all related to jaundice. There were also multiple highlighted words related to respiratory symptoms, such as *gas* with 18 mentions, *bicarb* having 9 mentions, and 3 mentions for *PCO_2_* Although no surgery plan was considered for the patient, the word surgery was present in the suffix, that is, this is an *out of context* prediction.

In Multimedia Appendix 2, we examined how early our algorithm can predict concept occurrences. In particular, in TP cases, we calculated the time from the prefix’s end to the suffix’s beginning. For most concepts, the minimum times are almost 0 because there are suffixes that occur right after their prefixes. On average, our algorithm can predict concepts several days before their actual occurrences.

#### False-Negative Analysis

We presented the same evaluation on FN cases in Table 6. Although 53 bronchoscopies were accurately mentioned and performed, the FN had an additional concept mentioned in context (1 time) or mentioned but only considered and not performed (3 times). Colonoscopy appeared more in the FN group with 21 colonoscopies mentioned and performed but had a high quantity of concepts mentioned in context (5) or mentioned but only considered and not performed (13). The surgery group also mentioned and performed 154 concepts; however, similar to Table 5, it has a significant number of predictions made out of context (8) or mentioned but only considered and not performed (42). The refractive surgery enhancement concept had the lowest ratio of concepts accurately mentioned and performed (48) to those mentioned out of context (21) or mentioned but only considered and not performed (14). Overall, 24.8% of FN cases are unimportant because of being out of context or not being performed by physicians.

**Table 6:** Expert evaluation of false negative predictions using 4 fine-grained categories (for instance, surgery was not predicted to be in suffix, and it appears in the suffix).

Concept	Mentioned and performed	Concept mentioned, but not needed for prediction	Mentioned but only considered by physician, not performed	Mentioned, but out of context
Bronchoscopy	49	0	3	1
Cardiac surgery	40	0	6	0
Colonoscopy	26	0	13	5
Craniotomy	23	0	2	1
Dialysis procedure	46	0	6	1
Refractive surgery enhancement	48	0	14	23
Surgery	154	0	43	9

## Discussion

### Principal Findings

Our results show that when applied to clinical concept prediction in ICU patients, symmetric interpatient distance measures are more robust in terms of *F* measure, sensitivity, and precision. Furthermore, antisymmetric interpatient distance measures performed the best in terms of specificity. Hence, antisymmetric interpatient distance measures are more conservative when predicting future medical concepts, as explained by their high confidence thresholds and high levels of specificity, whereas symmetric interpatient distance measures observe a 10% gain in precision and sensitivity over antisymmetric measures. Thus, symmetric interpatient distance measures are more predictive of future medical concepts. Overall, the APL_SYM performed the best.

We further evaluated the clinical value of the predictions. Our medical student and physician authors manually examined the TP and FN predictions of 16 important concepts. We found that 86.9% (353/406) of TP predictions are performed later, and only 4.7% (19/406) of the cases are totally out of context. This early concept prediction capability implies substantial impacts, such as avoiding potential high-risk events and improving patient outcomes at lower costs. On the other hand, our algorithm missed 513 FN cases, but 24.7% of them were clinically unimportant. Specifically, these missed concepts do appear in the patient suffixes but are out of context, or not needed, or not performed by the physician.

As an example of an application of the proposed methods in a real setting, we considered using these methods to periodically automatically predict the estimated number of patients in a hospital that will require bronchoscopy. This may allow for better resource planning.

### Limitations

We recognized that in its current form, our system is not sufficiently accurate for deployment. In particular, concern arises when giving a patient or their family access to our proposed methods—incorrectly predicting an undesired concept may incur unneeded stress and anxiety. In this regard, we may calibrate the confidence parameters to achieve higher precision and have an expert manually select the set of concepts that are appropriate to present to patients. As an example of a potential application, such a controlled prediction module could be deployed in a patient portal of a health insurance company, where a patient can already view his or her EHR.

From a medical perspective, ICUs are often numerically oriented with vital signs, pressure readings, laboratory values, and ventilator readings. Furthermore, ICUs move at a fast pace, and hence, using the granularity of ward transfers is perhaps too broad in the ICU setting. Therefore, our proposed methods will most likely achieve different results in a primary care or outpatient setting. An interesting analysis would be to compare long-term predictions in the outpatient setting with near-term predictions in the ICU setting.

However, the MIMIC database is one of the few, if only publicly available databases of EHRs that are rich in both clinical notes and temporal data. Clinical notes enable a rich collection of clinical concepts and hence allow for the prediction of a broad range of clinical concepts. *For example*, an EHR database containing only disease classifications will represent diabetes but will fail to represent insulin; hence, insulin cannot be predicted. Furthermore, temporal data allow us to sort medical concepts into prefixes and suffixes.

Another medical limitation is that we did not weigh concepts based on their clinical importance. For example, the concept of *cardiac arrest* is more important in terms of similarity and predictive value than the concept of *coughing*. Moreover, the importance of a clinical concept depends on its application and domain. Furthermore, we need to assess the accuracy required for our system to be useful to patients, clinicians, and researchers. This accuracy requirement could be assessed through user evaluations.

From a technical perspective, a key limitation is the assumption that MetaMap correctly identifies all concepts written in a clinical note. MetaMap has achieved reasonable precision and recall values (80% and 79%, respectively) when identifying medical concepts from clinical notes [34]. Given the raw text of a clinical note, this assumption is clearly invalid because of abbreviations in the clinical note and errors generated by MetaMap. We address abbreviations by using a manually crafted list of medical abbreviations common to clinical notes; thus, potential errors caused by ambiguities because of common abbreviations were minimized. Furthermore, we argue that errors generated by MetaMap are a natural language processing problem, which is beyond the scope of this study. MetaMap limitation also holds with any other automatic extraction tool. To mitigate this, our physician author manually evaluated the clinical significance of TP predictions for a subset of interesting concepts.

Another technical limitation is that we evaluated our algorithm strictly, in that we only accepted predictions that exactly predicted the corresponding concept. *For example*, if we predicted *cancer* when the actual concept was *breast cancer*, then our prediction of cancer would be marked as an FP, when our prediction was semantically relevant. Hence, including semantically similar concepts, either through is-a (ISA) ancestors or other semantic relations, has the potential to increase the accuracy of our algorithm while remaining relevant to clinical decision support.

### Conclusions

In this paper, we studied the problem of predicting future medical concepts in a patient’s EHR. The key idea of our method was to find patients with similar EHR prefixes using various interpatient similarity measures and then predict medical concepts that have high confidence in EHR suffixes of those patients. Our results showed that this is a promising approach to predict possible future concepts in a patient’s EHR. Of the multiple symmetric and antisymmetric interpatient similarity measures, the APL_SYM achieved the highest accuracy in our evaluation. We further evaluated the predictions of 16 important concepts manually and found that 86.9% of TP predictions are performed later. These initial results indicate that predicting a patient’s future medical concepts is feasible.

## Appendix

Multimedia Appendix 1 Prediction performance results for important concepts selected by our physician author. We do not count the cases that a predicted concept occurs in both patient’s prefix and suffix.

Multimedia Appendix 2 Time from our algorithm prediction to the actual occurrence of the concepts in suffix for true positive cases (The time is formatted as dd hh:mm:ss, where dd is dropped if the time is less than a day).

## Abbreviations

APL: average path length
APL_SYM: symmetric average path length
BoC: bag-of-concept
CA: common ancestor
DAG: directed acyclic graph
EHR: electronic health record
FN: false-negative
FP: false-positive
ICU: intensive care unit
MIMIC: Multiparameter Intelligent Monitoring in Intensive Care
SNOMED-CT: systemized nomenclature of MEDical clinical terms
TN: true-negative
TP: true-positive
UMLS: unified medical language system
