# Supplementary material for: Prediction of Medical Concepts in Electronic Health Records: Similar Patient Analysis
Source: JMIR Med Inform. 2020 Jul 17;8(7):e16008. doi: 10.2196/16008 (PMC7395257; doi:10.2196/16008)
Supplement: Multimedia Appendix 1 [file medinform_v8i7e16008_app1.docx]

| Concept | TP (Correctly predicted mention in suffix) | FP (Incorrectly predicted mention in suffix) | TN (Correctly predicted no mention in suffix) | FN (Incorrectly predicted no mention in suffix) | Sensitivity | Specificity |
| --- | --- | --- | --- | --- | --- | --- |
| Bronchoscopy | 63 | 948 | 2330 | 53 | 54.3% | 71.1% |
| Cardiac surgery procedure | 5 | 118 | 3267 | 46 | 9.8% | 96.5% |
| colonoscopy | 1 | 46 | 3306 | 44 | 2.2% | 98.6% |
| Craniotomy | 12 | 87 | 3259 | 26 | 31.6% | 97.4% |
| Dialysis procedure | 54 | 926 | 2355 | 53 | 50.5% | 71.8% |
| Refractive surgery enhancement | 14 | 157 | 3180 | 85 | 14.1% | 95.3% |
| Surgery | 257 | 867 | 1290 | 206 | 55.5% | 59.8% |
