# Supplementary material for: Prediction of Medical Concepts in Electronic Health Records: Similar Patient Analysis
Source: JMIR Med Inform. 2020 Jul 17;8(7):e16008. doi: 10.2196/16008 (PMC7395257; doi:10.2196/16008)
Supplement: Multimedia Appendix 2 [file medinform_v8i7e16008_app2.docx]

| Concept | Min | 25 Percentile | Median | Average | 75 Percentile | Max |
| --- | --- | --- | --- | --- | --- | --- |
| Bronchoscopy | 0:00:00 | 0:04:15 | 1d 2:0:30 | 3d 4:25:10 | 4d 9:44:30 | 27d 18:17:0 |
| Cardiac surgery procedure | 0:01:00 | 21:14:00 | 1d 4:46:0 | 3d 18:41:0 | 1d 10:23:0 | 15d 9:1:0 |
| colonoscopy | 0:02:00 | 0:02:00 | 0:02:00 | 0:02:00 | 0:02:00 | 0:02:00 |
| Craniotomy | 0:00:00 | 0:01:15 | 8:33:30 | 2d 3:13:42 | 3d 18:27:45 | 8d 13:5:0 |
| Dialysis procedure | 0:00:00 | 0:02:00 | 1d 19:31:0 | 6d 13:50:39 | 11d 2:0:0 | 45d 11:55:0 |
| Refractive surgery enhancement | 0:00:00 | 0:02:00 | 13:15:00 | 7d 10:59:20 | 13d 9:51:30 | 31d 14:11:0 |
| Surgery | 0:00:00 | 0:01:00 | 4:33:00 | 3d 13:40:34 | 3d 19:32:0 | 48d 3:24:0 |
